# Magnetic frustration induced large magnetocaloric effect in the absence of long range magnetic order

**DOI:** 10.1038/s41598-017-07459-3

**Published:** 2017-08-04

**Authors:** Santanu Pakhira, Chandan Mazumdar, R. Ranganathan, Maxim Avdeev

**Affiliations:** 1 Condensed Matter Physics Division, Saha Institute of Nuclear Physics, 1/AF, Bidhannagar, Kolkata, 700 064 India; 2 Bragg Institute, Australian Nuclear Science and Technology Organisation, Locked Bag 2001, Kirrawee DC, New South Wales 2232 Australia; 3 School of Chemistry, The University of Sydney, Sydney, NSW 2006 Australia

## Abstract

We have synthesized a new intermetallic compound Ho_2_Ni_0.95_Si_2.95_ in a single phase with a defect crystal structure. The magnetic ground state of this material found to be highly frustrated without any long range order or glassy feature as investigated through magnetic, heat capacity and neutron diffraction measurements. The interest in this material stems from the fact that despite the absence of true long range order, large magnetocaloric effect (isothermal magnetic entropy change, −ΔS_M_ ~ 28.65 J/Kg K (~205.78 mJ/cm^3^ K), relative cooling power, RCP ~ 696 J/Kg (~5 J/cm^3^) and adiabatic temperature change, ΔT_*ad*_ ~ 9.32 K for a field change of 70 kOe) has been observed which is rather hard to find in nature.

## Introduction

Magnetocaloric effect is a thermodynamical phenomenon in which change in material temperature occurs due to application of magnetic field under adiabatic condition. Magnetocaloric materials, working on the principle of magnetic refrigeration, are one of the most energy efficient and environment friendly replacement of those conventional systems based on gas compression/expansion technique^1–6^. In general, the compounds which exhibit large change in magnetic entropy, adiabatic temperature and cooling power, are considered as large MCE materials. Large MCE near room temperature is important for household purpose^7^, but for liquefaction of hydrogen, helium and space technology application^8^, low temperature region is also very important. A major goal in this emerging research area is to find new materials that exhibit large MCE and are capable to operate in different temperature ranges, suitable for corresponding application.

In most practical purposes sub-Kelvin temperatures are achieved through adiabatic demagnetization of paramagnetic salts^9^ or magnetic garnets^10^, but none of them is metallic. An ideal magnetocaloric material preferred to be metallic and non-superconducting in nature for better heat conduction and easy machining. The compound also should not degrade over time, *i.e*., the compound must be stable enough at the ambient condition. To overcome this problem, investigation of MCE was initially focused on metallic ferromagnetic materials around the ferromagnetic Curie temperature^11–13^. Later on it has been found that many antiferromagnetic metallic systems undergoing field induced ferromagnetism or metamagnetic transition also exhibit large MCE^14, 15^. However, in most cases this type of magnetic transition is usually accompanied by thermal and (or) magnetic hysteresis, which is disadvantage for application purpose. Another promising alternative material to exhibit large MCE can be metallic materials having infinitely degenerate magnetically frustrated ground states. With increase in magnetic field, degeneracy in the ground state tend to be lifted causing frustrated magnetic moments to polarize in the field direction. This also results in large magnetic entropy change. Recently, few theoretical predictions have also been made to obtain large value of MCE in frustrated magnetic systems^16–19^. Quite a few experimental supportive results for such theoretical prediction have been obtained till now^20–22^. Magnetic frustration are also known to enhance the barocaloric effect as well^23^. However, all the bulk materials so far reported to exhibit such frustrated ground state found to coexist with long range magnetic order. As a result, the exact role of magnetic frustration on the enhancement of MCE is difficult to determine. Large MCE material that show neither the long range magnetic ordering nor even the spin freezing behaviour is rather hard to find in nature.

In hexagonal ternary intermetallic compound *R*_2_ *TX*_3_, where *R* = rare-earth element, *T* = transition metal and *X* = Si, Ge, In, *etc*, only *R* ions generally carry the magnetic moment^24–26^. Since *T* and *X* ions are randomly distributed in the *2d* Wyckoff position, the local environment of *R* ions varies randomly. In the presence of antiferromagnetic interaction, such geometry may result in geometrical frustration^27^. Additionally, since the ratio of lattice parameters (*c/a*) approaches to be close to unity, one also expects to get strong frustration when nearest-neighbour exchange interaction (J_NN_) and next-nearest-neighbour exchange interaction (J_NNN_) are of opposite signs^28^. In this work, we show that Ho_2_Ni_0.95_Si_2.95_ forms in single phase only in defect structure and exhibits large MCE over a wide temperature range in the absence of any true long range magnetic order.

## Results and Discussions

**Figure 1:**
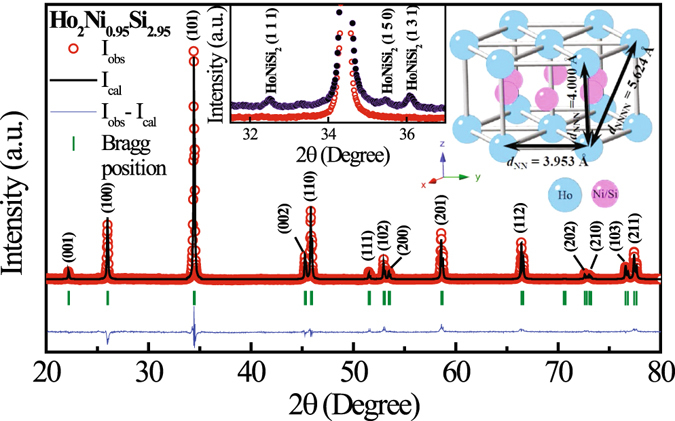
Room temperature XRD pattern of Ho_2_Ni_0.95_Si_2.95_ along with full Rietveld refinement. Inset shows the presence of HoNiSi_2_ type of secondary phase in Ho_2_NiSi_3_ (violet), while Ho_2_Ni_0.95_Si_2.95_ (red) form in single phase. Crystal structure of Ho_2_Ni_0.95_Si_2.95_ is also displayed marked with nearest-neighbour distances.

The room temperature X-ray diffraction (XRD) pattern of full stoichiometric Ho_2_NiSi_3_ found to contain minor (<10% of 100% peak) additional peaks of HoNiSi_2_ (Inset: Fig. 1). The phase purity could not be improved even on annealing. Single phase material however could only be obtained in defect structure Ho_2_Ni_0.95_Si_2.95_ (Fig. 1). The lattice parameters obtained are *a* = 3.953(2)Å and *c* = 4.000(1)Å (space group *P6/mmm*). Interestingly, we found that the *c/a* ratio close to unity, suggesting that nearest-neighbour (NN) and next-nearest-neighbour (NNN) distances for Ho ions are quite comparable. The crystal structure remains conserved down to 15 K, the lowest measurable temperature at our X-ray diffractometer. Neutron diffraction (ND) result (discussed later) suggests that the crystal structure does not change even at 1.5 K.

**Figure 2:**
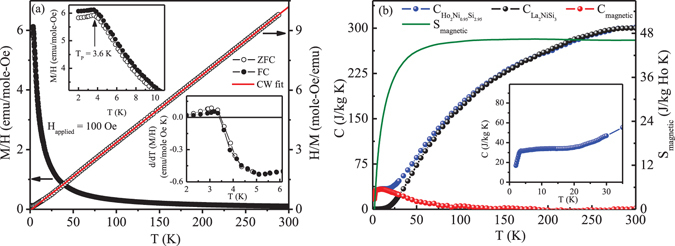
(**a**) *χ*(M/H) - T under ZFC and FC conditions (left panel) and *χ* ^−1^(H/M) - T (right panel) at H = 100 Oe. Upper inset: expanded low temperature region. Lower inset: Temperature derivative of *χ* in ZFC and FC conditions. (**b**) Temperature dependence of heat capacity of Ho_2_Ni_0.95_Si_2.95_, La_2_NiSi_3_, magnetic contribution (C_magnetic_)[left panel] and temperature dependence of magnetic entropy (S_magnetic_)[right panel]. Inset: expanded low temperature region of heat capacity for Ho_2_Ni_0.95_Si_2.95_.

The temperature dependence of dc magnetic susceptibility (*χ* = M/H) under zero-field-cooled (ZFC) and field-cooled (FC) protocols at 100 Oe applied magnetic field show a peak-like structure at T_P_ = 3.6 K in both the protocols [Fig. 2(a)]. The temperature derivative of dc magnetic susceptibility exhibit a crossover from negative to positive value, which is commonly considered to be characteristic of antiferromagnetic transition. The peak at T_P_, however, appear to be quite weak in nature, as *χ*(0)/*χ*(T_P_) found to be close to unity. Generally, in a polycrystalline material with collinear Heisenberg antiferromagnetic arrangement, one expects *χ*(0)/*χ*(T_P_) to be 2/3^29^. The relatively large value of *χ*(2 K)/*χ*(T_P_) ~ 0.985 in Ho_2_Ni_0.95_Si_2.95_ suggests that the magnetic spin structure, if any, would be of canted nature having strong ferromagnetic component^30^. Signature of ferromagnetic interactions could also be found from the positive value of paramagnetic (PM) Curie-Weiss temperature (*θ*_CW_ = 1.8 K) estimated from the inverse susceptibility in the paramagnetic region (60–300 K). In an ideal antiferromagnetic system, one would expect −*θ*_CW_ ~ T_N_ and should be even lower for geometrically frustrated systems. The presence of ferromagnetic interactions in an antiferromagnetic compound generally brings the value of *θ*_CW_ towards zero or even positive, depending on the strength of ferromagnetic interaction in the system. Below 60 K, inverse susceptibility devites from linearity, which may be due to growth of short range magnetic interactions in the system or due to crystalline electric field effect. The effective magnetic moment (*μ*_*eff*_) estimated to be 10.8 *μ*_*B*_/Ho^3+^ ion which is slightly larger than that of free Ho^3+^ ion (10.6 *μ*_B_). The slightly larger value of *μ*_*eff*_ in the compound may originate from the polarization of the conduction electron of Ni ion, or may be due to reduction in moment density, which generally found in frustrated magnetic systems^27^.

The origin of competing ferromagnetic and antiferromagnetic exchange interaction strength may be found in the crystal structure of the compound. In rare-earth (*R*) based intermetallic compounds exchange interaction between the *R* ions is of RKKY type and exchange interaction strength (J_*ex*_) depends on the inter-ionic distances (d) as, J_*ex*_ ~ 1/*d* ^3^. Since the ratio of the lattice parameters (*c/a*) of Ho_2_Ni_0.95_ Si_2.95_ is of the order of 1.01 (Fig. 1), the nearest-neighbour rare-earth ion distance [*d*_NN_ = *a* = 3.953(2)Å] is comparable to next-nearest-neighbour distance [*d*_NNN_ = *c* = 4.000(1)Å]. As a result, the nearest-neighbour exchange interaction (J_NN_) and next-nearest-neighbour exchange interaction (J_NNN_) found to be of comparable strength. Since the third nearest-neighbour rare-earth ions are placed relatively further away [*d*_NNNN_ = 5.624(2)Å], its contribution to the exchange interaction strength (J_NNNN_) is relatively quite small. It is therefore quite plausible that J_NN_ and J_NNN_, which are nearly of equal strength but of opposite sign, might be responsible for the competing nature of ferromagnetic and antiferromagnetic interactions in this compound.

The signature of long range magnetic ordering however found to be absent in the heat capacity measurement down to 2 K. It shows only a broad anomaly in the temperature range 3–25 K, with a rather sharp drop below 3 K [Fig. 2(b)]. The magnetic contribution (C_magnetic_) of molar heat capacity has been calculated by subtracting heat capacity data of isostructural stoichiometric La_2_ NiSi_3_ after appropriate lattice volume correction. The magnetic contribution shows a broad hump in the temperature range 3–25 K. Similar broad hump in heat capacity data generally found in systems having frustrated short range magnetic ground state^31–33^. The magnetic entropy (S_magnetic_) at T_P_ is found to be only about 60% of R*ln*2, suggesting the absence of true long range ordering in the system. The magnetic entropy value reaches saturation value [R *ln*(2*J* + 1) = R*ln*17, with *J* = 8] around 60 K, due to presence of spin fluctuation and presence of short range magnetic correlation up to such higher temperature^34^. This is in agreement with the deviation of Curie-Weiss law of inverse susceptibility below 60 K.

**Figure 3:**
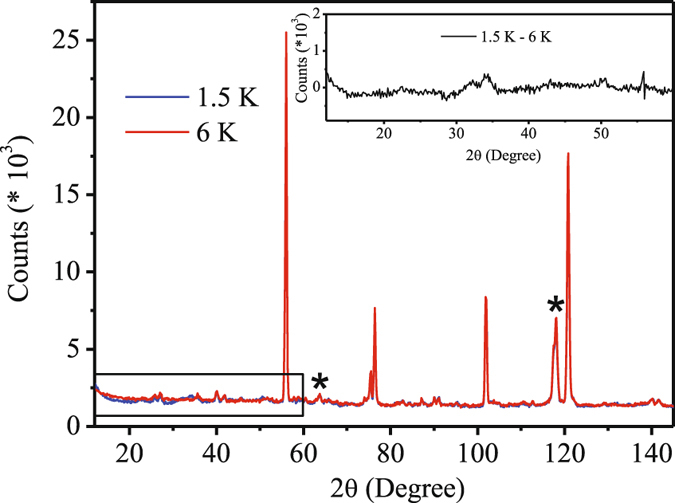
Zero field neutron diffraction pattern for T = 1.5 K (blue), 6 K (red) and their difference (inset). Al sample container contribution in diffraction pattern is marked with asterisk (*).

Neutron diffraction experiments for this polycrystalline compound were carried out at different temperatures, above and below T_P_ (=3.6 K), to look for the possible arrangement of magnetic spin structures (Fig. 3). The data collected at 1.5 K showed magnetic peaks which were much weaker than those expected for Ho^3+^ with *gJ* = 10 *μ*_*B*_ and much broader than the nuclear diffraction peaks. The weak intensity of magnetic peaks did not allow to fully determine the magnetic structure. However, the correlation length was estimated from the width of magnetic peaks using the Scherrer formula which yielded the value of ~35 Å. This is the same order of magnitude previously found for magnetically frustrated materials with short range magnetic correlations^35–37^. Thus, both the neutron diffraction as well as heat capacity result confirms the absence of true long range ordering in Ho_2_Ni_0.95_Si_2.95_. In all practical purpose, the system thus appear to be in the magnetically frustrated state. Such frustration might have originated due to random distribution on Ni/Si along with strongly competing exchange interaction originating for *c*/*a* ~ 1.

**Figure 4:**
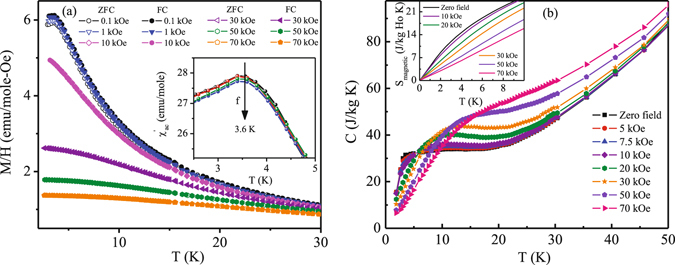
(**a**) *χ* - T for different magnetic field changes. Inset: expanded low temperature region of real part ac susceptibility at different frequencies. (**b**) Heat capacity as a function of temperature measured under different external magnetic fields, H. Inset: S_magnetic_ - T for different H.

A magnetically frustrated state coupled with random disorder is generally known to be conductive of spin or cluster glass behaviour^27^. The estimated low values of relaxation time constant (~120 sec) cast doubt about the presence of any spin glass freezing behaviour in this compound. The peak in the real part of ac susceptibility data around 3.6 K is frequency independent, which further confirms the absence of any glassy interaction [Inset: Fig. 4(a)]. The compound also does not show any magnetic memory effect which is generally exhibited by glassy systems. Thus, surprisingly, despite having strong disorder density, Ho_2_Ni_0.95_Si_2.95_ does not show any glassy magnetic feature.

One of the main feature of magnetically frustrated systems is that their ground states are highly degenerate in nature. On application of magnetic field, moments try to align themselves along the field direction by lifting the ground state degeneracy. After a critical field strength (H_sat_) majority of the moments get aligned in the field direction making a unidirectional spin alignment. The value of the critical field strength depends on the degree of frustration present in the system. Field dependent dc magnetic susceptibility of Ho_2_Ni_0.95_Si_2.95_ [Fig. 4(a)] shows that the peak observed at T_P_ (=3.6 K) at low external fields vanishes above 10 kOe because of the polarization of the short range moments in the field direction. Further increase in applied magnetic field strength results in increasing ferromagnetic-like volume fraction. This behaviour is also reflected in the heat capacity measurement under various external fields [Fig. 4(b)]. The rather sharp drop in heat capacity observed below 3.6 K, as described earlier, appear to get broadened with the application of magnetic field similar to that found in other magnetically frustrated systems^31–33^. With the application of magnetic field, the magnetic entropy, estimated from the heat capacity result, gradually gets reduced in such frustrated magnetic system due to lifting of magnetic degeneracy [inset: Fig. 4(b)]. The rate of change of magnetic entropy with applied magnetic field exhibit a discrete change for field above 10 kOe. Such abrupt change in magnetic entropy is also an indicator of large magnetocaloric effect in this system that exhibit no long range magnetic order.

**Figure 5:**
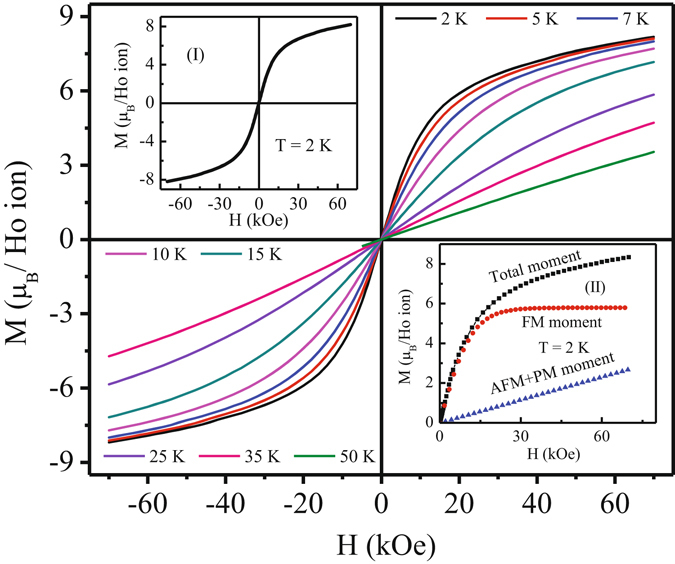
Field dependence of isothermal magnetization during field change 0 Oe → 70 kOe → −70 kOe → 70 kOe of Ho_2_Ni_0.95_Si_2.95_ at different temperatures. Inset (I) shows M-H at 2 K. Inset (II) shows M - H at 2 K along with extracted different types of magnetic volume fraction using equation: *M*(*H*) = *A*[*tanh*(*BH*)] + *CH*.

The field variation of magnetization, measured at 2 K, shows a linear behaviour for H ≤ 10 kOe, but tends to saturate at higher field (Fig. 5). The value of magnetic moment for 70 kOe field change is 8.18 *μ*_*B*_/Ho^3+^ ion, which is slightly smaller than the theoretical saturation value (10 *μ*_*B*_/Ho^3+^-ion, with *g* = 5/4 and *J* = 8). It is surprising that magnetically frustrated Ho_2_Ni_0.95_Si_2.95_ resulted such ferromagnetic like behaviour with large magnetic moment value, implying a strong modification of the competition of ferromagnetic and antiferromagnetic exchange interaction under the influence of magnetic field. The magnetic field dependence can thus be analysed considering a combined effect of ferromagnetic and antiferromagnetic isotherms as *M*(*H*) = *A*[*tanh*(*BH*)] + *CH*, where, *A*, *B* and *C* are fitting parameters. The first term in the equation corresponds to ferromagnetic volume fraction and origin of the linear term is the combining effect of antiferromagnetic and paramagnetic volume fraction. Such type of analysis have also been performed for earlier reported systems with ferromagnetic and antiferromagnetic interactions^38^. As seen from inset II of Fig. 5, moment of the ferromagnetic component saturates above an applied field value of 20 kOe. As temperature increases, the ferromagnetic like contribution weakens gradually. However, the M(H) value taken at a temperature (50 K) much higher than T_P_ (=3.6 K), still do not show linear behaviour expected in a truly paramagnetic system. This is in commensurate with our observation that inverse susceptibility deviates from linearity below 60 K. No magnetic hysteresis is observed even at the lowest temperature (2 K, inset I: Fig. 5).

**Figure 6:**
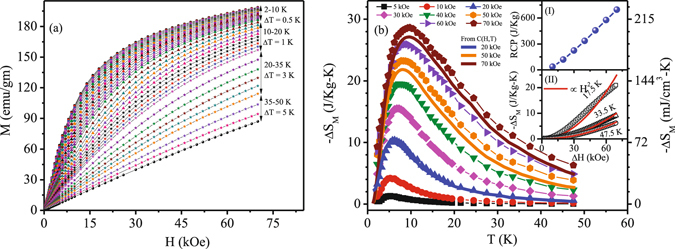
(**a**) M(H) at different temperatures during field change 0 Oe → 70 kOe. (**b**) Temperature dependence of isothermal magnetic entropy change (−ΔS_M_) at different field changes. Inset (I): Relative cooling power (RCP) as a function of applied field changes. Inset (II): H^2^ dependence of −ΔS_M_ at different temperatures.

**Figure 7:**
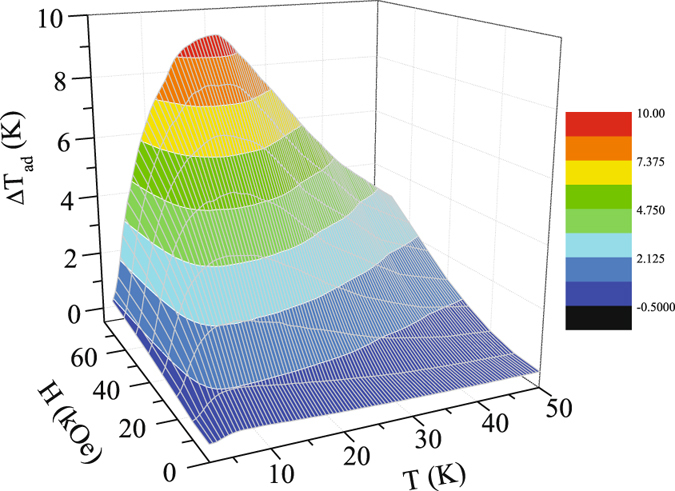
Temperature and magnetic field evolution of adiabatic temperature change (ΔT_*ad*_).

The magnetocaloric effect of Ho_2_Ni_0.95_Si_2.95_ have been estimated from the isothermal magnetization [Fig. 6(a)] and zero field heat capacity data, by estimating magnetic entropy changes $[{\rm{\Delta }}{{\rm{S}}}_{{\rm{M}}}={\int }_{{{\rm{H}}}_{1}}^{{{\rm{H}}}_{2}}(\frac{{\rm{\partial }}M}{{\rm{\partial }}T}){\rm{d}}{\rm{H}}]$, relative cooling power $[{\rm{RCP}}={\int }_{{{\rm{T}}}_{1}}^{{{\rm{T}}}_{2}}{\rm{\Delta }}{{\rm{S}}}_{{\rm{M}}}{\rm{d}}{\rm{T}}]$ and adiabatic temperature change [ΔT_*ad*_ = [T(S, H) − T(S, 0)]_S_]. All the three parameters found to be quite large for this compound. For example, the maximum value −ΔS_M_ is found to be 28.65 J/Kg K (~205.78 mJ/cm^3^ K) and 23.25 J/Kg K (~167 mJ/cm^3^ K) for a field change of 70 kOe and 50 kOe, respectively [Fig. 6(b)]. Even for a low field change of 20 kOe, the value of −ΔS_M_ is 10.5 J/kg K (~75.42 mJ/cm^3^ K), being very beneficial for application purpose. The basic nature of −ΔS_M_(T) estimated from heat capacity measurements are quite similar, except for a minor difference in absolute magnitude. These values are comparable or even larger than those reported for most of the potential magnetic refrigerant intermetallic materials exhibiting ferromagnetic ground state^13, 39, 40^ or antiferromagnetic ground state with metamagnetic transition(s)^14, 15, 41, 42^ in the cryogenic temperature region. Observation of such large value of −ΔS_M_ is extremely rare in intermetallic compounds having frustrated ground state with no true long range ordering. Additionally, the large value of −ΔS_M_(T) coupled with its asymmetric spread over a wide temperature range makes the RCP values very high as well. The calculated RCP values for Ho_2_Ni_0.95_Si_2.95_ are 460 J/Kg (~3.3 J/cm^3^) and 696 J/Kg (~5 J/cm^3^) at field change of 50 kOe and 70 kOe, respectively [inset I: Fig. 6(b)], which are one of the largest value of RCP reported for good refrigerant material around this temperature scale^13, 15, 39, 40, 43, 44^. It may be noted here that in case of long range magnetic order −ΔS_M_(T) appears to be symmetric around the Curie temperature, while asymmetric spread are seen primarily in case of spin fluctuations^45^ or spin flop transitions^46^. In the paramagnetic region, theoretical calculation yields −ΔS_M_ ~ H^2^/2T^2^, where H is the applied field and T is the corresponding temperature. At low temperature, −ΔS_M_(H) deviates quite significantly from the H^2^ behaviour [inset II: Fig. 6(b)]. As temperature increases, the discrepancy to H^2^ behaviour decreases. However, even at 47.5 K, −ΔS_M_(H) still exhibit minor discrepancy with H^2^ behaviour, suggesting the system has not yet reached truly paramagnetic state which is in accordance with the magnetic susceptibility and heat capacity results described earlier. The short range magnetic correlations due to strong magnetic frustration of the ground state of Ho_2_Ni_0.95_ Si_2.95_ thus plays a key role in exhibiting giant RCP values. Similar to ΔS_M_ and RCP, the other important MCE parameter ΔT_*ad*_ also found to be quite high. The maximum values of ΔT_*ad*_ are 6.61 K and 9.32 K for a field change 50 kOe and 70 kOe, respectively [Fig. 7]. We thus found that the frustrated magnetic intermetallic compound Ho_2_Ni_0.95_Si_2.95_ exhibits very large values of all the three relevant parameters *viz*., −ΔS_M_, RCP and ΔT_*ad*_, required for a good magnetic refrigerant material.

To conclude, we found Ho_2_ Ni_0.95_Si_2.95_ is one of the extremely rare intermetallic compound having frustrated ground state with no true long range magnetic ordering that show large MCE. The relevant MCE parameter values are comparable or even larger than those reported for most of the potential magnetic refrigerant intermetallic materials exhibiting ferromagnetic ground state or antiferromagnetic ground state with metamagnetic transition(s) in the cryogenic temperature region. The absence of long range magnetic order indicates that the magnetic frustration is primarily responsible for large MCE in this compound. Such mechanism, although theoretically predicted earlier, had been rarely observed experimentally in any other systems.

## Methods

The polycrystalline samples were synthesized in arc furnace by melting appropriate amount of constituent elements (purity >99.99%) under inert (Ar) atmosphere using a water cooled Cu hearth. The ingot was re-melted several times, by flipping every time to promote volume homogeneity. The weight loss is less than 0.2%. X-ray diffraction (XRD) experiments were performed on the powdered as-cast sample using Cu-K_*α*_ radiation on a Rigaku TTRAX-III powder diffractometer having 9 kW power in the temperature region 15–300 K, for structural characterization. Full-Rietveld analysis of XRD data was carried out using FULLPROF package^47^. The magnetic measurements were carried out in a SQUID VSM (M/s Quantum Design, Inc., USA) and Ever Cool II VSM (M/s Quantum Design Inc., USA) in the temperature range 2 K–300 K and magnetic field up to 70 kOe. The heat capacity measurements were carried out in PPMS (M/s Quantum Design, Inc., USA) in the temperature range 2 K–300 K and magnetic fields up to 70 kOe. The neutron diffraction experiments were performed at ECHIDNA beam line in ANSTO, Australia at different temperatures.
